# Migrant tuberculosis patient needs and health system response along the Thailand–Myanmar border

**DOI:** 10.1093/heapol/czx074

**Published:** 2017-07-02

**Authors:** Naomi Tschirhart, Francois Nosten, Angel M Foster

**Affiliations:** 1 Faculty of Health Sciences, Interdisciplinary School of Health Sciences, University of Ottawa, 1 Stewart Street, Ottawa, ON, Canada K1N 6N5,; 2 Shoklo Malaria Research Unit, Mahidol-Oxford Tropical Medicine Research Unit, Faculty of Tropical Medicine, Mahidol University, PO Box 46, Mae Sot, Tak 63110, Thailand and; 3 Centre for Tropical Medicine and Global Health, Nuffield Department of Clinical Medicine, University of Oxford, Churchill Hospital, Oxford, UK

**Keywords:** TB, migrants, Thailand, Myanmar, health system

## Abstract

This article aims to identify how the health system in Tak province, Thailand has responded to migrants’ barriers to tuberculosis (TB) treatment. Our qualitatively driven multi-methods project utilized focus group discussions, key informant interviews, and a survey of community health volunteers to collect data in 2014 from multiple perspectives. Migrants identified legal status and transportation difficulties as the primary barriers to seeking TB treatment. Lack of financial resources and difficulties locating appropriate and affordable health services in other Thai provinces or across the border in Myanmar further contributed to migrants’ challenges. TB care providers responded to barriers to treatment by bringing care out into the community, enhancing patient mobility, providing supportive services, and reaching out to potential patients. Interventions to improve migrant access and adherence to TB treatment necessarily extend outside of the health system and require significant resources to expand equitable access to treatment. Although this research is specific to the Thailand–Myanmar border, we anticipate that the findings will contribute to broader conversations around the inputs that are necessary to address disparities and inequities. Our study suggests that migrants need to be provided with resources that help stabilize their financial situation and overcome difficulties associated with their legal status in order to access and continue TB treatment.

Key Messages

- Migrants in Tak province, Thailand experience barriers to tuberculosis (TB) treatment related to legal status, transportation, finances and unavailability of TB care in other jurisdictions.
- Two non-profit TB programmes have responded to treatment barriers by bringing care out into the community, enhancing patient mobility, providing supportive services, counselling patients and reaching out to potential patients.
- Interventions to improve migrant access and adherence to TB treatment necessarily extend outside of the health system and require significant resources.

## Introduction

Tuberculosis (TB) disproportionately impacts vulnerable populations. Individuals who experience poverty face an embedded disadvantage as being poor is associated with a higher risk of TB infection, active disease, delayed diagnosis, poor adherence, and fatality (Noyes and Popay 2007; Lönnroth *et al.* 2014). Concerns about lost earnings and transportation costs influence poorer patients’ decisions to seek treatment (Noyes and Popay 2007). Socio-economic status also influences adherence to TB treatment, as therapy is lengthy and patients may experience a reduction in income (Mauch *et al.* 2013; Naidoo *et al.* 2013; Paz-Soldán *et al.* 2013; Richter *et al.* 2014). Migrant populations experience this tension between their financial obligations and a need to continue TB treatment as well as additional difficulties related to legal and registration status (Xu et al. 2009; Bele *et al.* 2014; Shringarpure *et al.* 2016). The Stop TB Partnership’s Global Plan to end TB identifies migrants as a priority group that has limited access to TB treatment (Stop TB Partnership 2015). In recognition of the specific challenges migrants face when accessing healthcare, a global consultation identified the importance of migrant sensitive health systems that ‘consciously and systematically incorporate the needs of migrants into health financing, policy, planning implementation and evaluation’ (World Health Organization 2010 p. 61). Delivery of migrant sensitive care is enhanced through interpretation services, culturally informed care and programmes, and cultural support personnel such as community health workers (World Health Organization 2010). To date, literature on how providers respond to TB patients’ non-medical needs remains limited and little is known about how health systems respond to migrants’ needs, in particular(Noyes and Popay 2007; Richter *et al.* 2014).

The Thailand–Myanmar border is a region of increasing geopolitical importance. Separated by the Moei river, Tak province in western Thailand and Kayin state in eastern Myanmar share a distinctive mountainous topography and a long history of transnational migration. Recent peace agreements in Myanmar signaled the end of a decades-long conflict that had sent thousands of refugees across the border to Thailand. Improved political stability has spurred economic development and the Thai border town of Mae Sot is emerging as a significant economic centre. An estimated 300 000 registered migrants and 90 000 refugees live in Tak province; cross-border migrants live in Myanmar and traverse the border to seek care (Hemhongsa *et al.* 2008; Iemrod and Kavinum 2015). Along the border, healthcare is provided to migrants by Thai government hospitals and non-governmental organizations (NGOs). Migrants typically make less than the national daily minimum wage of 300 baht (USD 8) and individuals who lack the necessary documentation to remain legally in Thailand face difficulties travelling around the province and must try to avoid the police checkpoints which dot the landscape (Tschirhart *et al.* 2016a,b). Refugee and migrant populations living in Tak province are disproportionately burdened by TB (Hemhongsa *et al.* 2008; Iemrod and Kavinum 2015). Although TB treatment for undocumented migrants is not covered under Thailand’s universal healthcare coverage scheme, Thai government hospitals may give TB treatment on a case-by-case basis depending on funding. In 2014, a grant from the UK’s Department for International Development and the European Union provided funding for undocumented migrants’ TB treatment at five Thai government hospitals close to the border.

In this region NGOs established specific TB programmes to provide care for migrants who were not eligible for free healthcare from the Thai government hospitals or needed additional supportive services (Tschirhart *et al.* 2016a,b, 2017). World Vision Thailand (WVT) and the Shoklo Malaria Research Unit (SMRU) run community-based TB programmes. WVT’s TB programme operates in partnership with Mae Sot Hospital, the district government hospital, and is run through community health posts in migrant communities around Mae Sot. Community health volunteers (CHVs) provide basic health services and help arrange transport for patients who need to go to the hospital. The tradition of CHVs, individuals who provide basic health care, is well established among both Thai populations and migrant populations on both sides of the Thailand–Myanmar border (Lee *et al.* 2009; Kowitt *et al.* 2015). WVT’s programme provides care to foreign labour migrants who are residing in the greater Mae Sot area. Conversely, SMRU’s TB programme provides care to a wider population including refugees, cross-border migrants, and migrants living in Thailand. SMRU has developed a residential programme where patients come to stay at the treatment centre. The SMRU TB village sits on a hill surrounded by agricultural fields just outside of Wang Pha village on the Thai side of the Thailand–Myanmar border. Rows of one-room dwellings accommodate patients and accompanying family members are housed separately. Patients are provided shelter, medication, and food free-of-charge.

In addition to WVT and SMRU, Mae Tao Clinic (MTC) and Première Urgence – Aide Médicale Internationale (PU-AMI) also contribute to TB control among migrants. MTC, a well-known clinic that provides healthcare for migrants along the Thailand–Myanmar border, assists in the detection of a large number of TB cases and refers patients to SMRU for TB treatment. PU-AMI runs a TB programme in the refugee camp that is predominantly for refugees but also treats migrants who have gained access to the camp. As mobility in an out of the refugee camp is severely restricted, migrants who gain access to the camp often have a family connection and permission to enter which allows them to seek TB care from PU-AMI.

Our research project aimed to investigate access to TB treatment for migrants and refugees, TB surveillance, and health systems response. We have published our findings on treatment accessibility, surveillance, and TB control elsewhere (Tschirhart *et al.* 2016a,b, 2017). In this article we address the research question: how have community and non-governmental healthcare providers responded to treatment barriers? Drawing from focus group discussions (FGDs), key informant (KI) interviews and a CHV survey, we identify how the health system, inclusive of CHVs, medics, doctors, and programme administrators, has responded to barriers to TB treatment experienced by migrants in Tak province, Thailand. We define responsive actions as initiatives undertaken to address migrants’ needs. We examine responses by NGO TB programmes and CHVs, which seek to improve migrants’ access and adherence to TB treatment.

## Materials and methods

### Data collection

We conducted fieldwork for this research project in Tak province Thailand from July to October 2014. Our multi-methods project utilized FGDs, KI interviews, and a survey of CHVs to explore access to TB treatment from multiple perspectives. We have described our FGD and KI methodology in detail in previous articles (Tschirhart *et al.* 2016a,b, 2017).

#### Focus group discussions

With the assistance of Burmese and Karen language interpreters we conducted 11 FGDs with migrant and refugee TB, multidrug-resistant tuberculosis (MDR-TB), and TB-HIV patients to solicit information on barriers to treatment. Patient participants were undergoing treatment at three different treatment centres, namely a Thai government hospital, a clinic in the refugee camp and a TB village that provides care to migrants. We also held four FGDs with CHVs who were associated with a TB programme that provides supportive care.

#### KI interviews

We interviewed 13 KIs who were working with different organizations as TB treatment providers, programme administrators, and public health officials. In interviewing KIs we sought to gain insight into their experience and perceptions based on their work in TB control (Kelly 2010). Our interview guide explored perceived barriers to treatment for migrants and refugees as well as responsive actions. We asked KIs to identify changes that had been implemented in the previous two years as well as responsive actions aimed to improve treatment access.

#### CHV survey

In an effort to understand better CHVs’ contribution to TB control in Tak province we conducted a baseline survey of volunteers (*n* = 101) affiliated with a TB programme run by WVT. Our sample represents approximately half of the total number of CHVs associated with this programme. TB programme staff recruited CHVs in the peri-urban Mae Sot area. To be eligible for the survey CHVs had to be currently affiliated with a community health programme in Tak province, 20 years of age or older, sufficiently fluent in Thai, English, Karen or Burmese to complete the survey, affiliated with a TB programme, and willing to provide consent to participate. WVT staff collected data and read the survey to volunteers and wrote down the answers. Survey questions elicited information on CHVs’ contributions to TB treatment, referrals, and surveillance. The questionnaire is available upon request.

### Data analysis

We transcribed and translated all of the audio files from the FGDs and KI interviews into English and used NVivo 11 software to manage the data. We employed a hybrid deductive and inductive approach to thematic analysis and coded the data for pre-determined themes as well as those which were emergent and data driven (Boyatzis 1998). Our high level deductive themes of treatment barriers and responsive actions are integral to the study’s research aims; we included these in the FGD and KI interview guides. We simultaneously coded the data for themes, distinct explanatory constructs, and for codes, smaller units of analysis which help to explain the phenomenon (Boyatzis 1998; Guest *et al.* 2012). To further investigate perceived barriers to treatment we separated the perceptions of migrants, refugees, and KIs and explored the overlap and concordance (Tschirhart *et al.* 2016a). After discovering that migrants reported more difficulties accessing treatment than refugees, we mapped responsive actions onto migrants’ barriers to care.

To analyse the CHV survey data we manually entered the survey responses into Excel and then conducted descriptive statistical analyses using SPSS software. We used Fisher’s exact test to examine the relationship between categorical variables and look for statistical significance. However given the baseline nature of this survey and the large number of categorical variables, we did not find any meaningful statistical relationships. Thus, we report the descriptive frequencies in this article.

### Ethics

All participants provided consent prior to participating. We received ethics approval for this study from the Health Sciences and Sciences Research Ethics Board at the University of Ottawa (File No. H02-14-08), the Oxford Tropical Research Ethics Committee at the University of Oxford (538-14) and the Tak Provincial Public Health Office (TK 1/2557).

## Results

### Barriers to treatment

Migrants identified legal status and transportation difficulties associated with not having the appropriate documentation as the primary barriers to seeking TB treatment (Tschirhart *et al.* 2016a). Lack of financial resources, in addition to difficulties locating appropriate and affordable health services in other provinces of Thailand and across the border in Myanmar, contributed to the challenges faced by migrants with TB (Tschirhart *et al.* 2016b). In a previous article we reported the barriers and enabling factors for migrants and refugees seeking TB treatment (see Table 2, Tschirhart *et al.* 2016a). Here we have provided a list of migrants’ major barriers to treatment along with the responsive actions in Table 1. In our KI and FGD data, we identified responsive actions at the individual and programmatic levels that sought to improve migrants’ access and adherence to TB treatment.

**Table 1.** Barriers for migrants seeking TB treatment and responsive actions

Thematic domain	Barriers	Responsive actions	Actor
Financial	Family/work responsibilities	Accommodation for family members at the TB village	SMRU
		Outpatient care	WVT and SMRU
	Money problems	Community fund	Migrant community members
		Financial support	Individual health care providers
		Food and accommodation	SMRU
	Housing	Accommodation at the TB village	SMRU
TB Health services	Language	Provide free TB care in languages migrants understand	SMRU, WVT in collaboration with Mae Sot Hospital and PU-AMI
	Treatment cost		
	Services not available		
	Time to diagnosis	Improved diagnostics	SMRU
	Duration	Outpatient care	WVT/SMRU
	HIV co-infection and stigma	Expand treatment eligibility to include family members	SMRU
Transport	Travel restrictions	Deliver medication	SMRU
	Police/documents	Provide travel documents	SMRU
	Travel cost	Organize transport	SMRU and WVT
Legal status	Undocumented	Policy change on migrant worker registration and healthcare scheme enrollment	National Government of Thailand
		Provide travel documents	SMRU
Patient beliefs and behaviours	Delayed care seeking	Increased screening	WVT and SMRU
	Limited knowledge of TB and health system	Information dissemination by health volunteers	WVT
		Links with migrant health clinics	SMRU
		Counselling	SMRU
	Mobility	Counselling	SMRU and WVT
		Contract	WVT
Psychosocial support	No caregiver	Psychosocial activities	SMRU
		Encouragement	WVT CHVs

**Table 2.** CHV demographic characteristics

	*N* (%)
**Total**	101 (100%)
**Demographic characteristics**	
Gender	
Male	29 (29%)
Female	70 (70%)
Missing	2 (2%)
Age	
<20–29	22 (22%)
30–49	62 (61%)
50>59	17 (17%)
Legal status	
Undocumented migrant	30 (30%)
Documented migrant	61 (60%)
Thai citizen	1 (1%)
Prefer not to answer	3 (3%)
Missing	6 (6%)
Languages spoken^a^	
Karen	17
Burmese	94
Thai	13
Other	2
Missing	3
Length of time as a CHV	
<6 months <12 months	20 (20%)
1 < 2 years	23 (23%)
2 or more years	55 (54%)
Missing	3 (3%)

a Percentages are not reported for multiple answer questions.

### Responsive actions

Based on both the FGDs and the KIs, we identified two organizations that had adapted their services and service delivery in response to difficulties experienced by migrant patients. WVT and SMRU responded to migrants’ needs by simultaneously bringing care out into the community, enhancing patient mobility, providing supportive services, and reaching out to potential patients.

#### Community-based care delivery

Migrants who participated in our research project were primarily daily waged labourers. In seeking TB treatment they lost income for the days they were unable to work. Our participants, many of whom had minimal savings to offset the impact of lost wages, described the difficulty of balancing their own need for TB care with their familial financial responsibilities. A cross-border male migrant TB patient explained, ‘In my house I’m the main provider so it’s a problem for my family when I’m away for treatment. I worry for my family. Even if it’s not easy, I can’t do anything about it because I was sick and needed to seek treatment in order to get well*.’*

WVT and SMRU have responded to migrant patients’ financial challenges by adapting service delivery to bring TB treatment out of the clinic and into the community. In the WVT programme, patients initially receive TB treatment from a doctor at the Thai government hospital and subsequently WVT CHVs provide TB medication to the patient through the community health posts. A volunteer described,

It’s ok because the volunteer health worker will come and give medication at your home every day and you can still continue your work. Usually the volunteer health worker also has their own work so they’ll give one month of medication at the same time or sometimes for one week because they can’t come every day. World Vision makes it easy for us, they bring medication at home so we don’t have to go to another hospital or clinic like in the past.

In response to patients’ need to support their families, SMRU began offering community-based outpatient care to individuals who had successfully completed two months of treatment at the TB village and had shown good adherence. A KI explained the rationale behind this change,

To promote adherence, before we persuaded all of the patients to stay in our TB village before we completed treatment. But now we learned from them that they have a really hard life and they have families that rely on them. If we keep them over here, if we are very strict, they will run away. So some of them who have finished two months of treatment and have shown good adherence, if they are willing and fit to go, we let them go. But we have more logistical work to do to provide transport for their follow-up appointment or to follow-up with Mae Sot Hospital or Mae Tao Clinic for their drug supply.

SMRU also has health workers and home visitors working in the community who can help to follow-up with patients and provide medication in circumstances where migrants are unable to travel for follow-up treatment. In addition, SMRU opened another TB village just across the border from Tak province in Koko, Myanmar where migrants can seek care without having to cross the border into Thailand.

#### Enhancing patient mobility

As we have detailed elsewhere patients indicated that their ability to travel was closely related to their legal status and whether they had the correct documentation required to travel freely within Tak province (Tschirhart *et al.* 2016a,b). KIs who were working as health care providers also acknowledged migrants’ mobility challenges (Tschirhart *et al.* 2016a). WVT and SMRU responded by providing transportation to help patients reach treatment. A female migrant patient with MDR-TB described, ‘I have no legal status so whenever I pass the check point then I had to pay the police. But now I don’t have to pay and be afraid of the police because now SMRU arranges everything for me.’

To further enhance patient mobility, patients are provided with a treatment card which they can attempt to use to negotiate with law enforcement as evidence of a legitimate reason for travel. A KI explained, ‘We try to negotiate through our logistics team with local authorities, to explain that we are providing treatment like this, so when he comes to collect the drugs please let him go if he can show this card. Meaning that they have a treatment card and adherence check documentation.’

#### Providing supportive services

Migrant TB patients who participated in our study had a limited amount of available financial resources to offset the indirect costs associated with TB treatment (Tschirhart *et al.* 2016a,b). Participants indicated that food, accommodation, and psychosocial support services helped them to access and continue treatment (Tschirhart *et al.* 2016a). Through residential care at the SMRU TB village, migrants receive meals and housing for themselves and their family members. A KI explains the decision to extend housing to family members, ‘For male TB patients, not being able to earn money to provide for their family is a big challenge. This is especially true for migrants. So that’s why we have to keep their family members along with the patient at the clinic.’ Although participants did not receive cash incentives for completing treatment, several patients indicated that care providers gave them pocket money to assist with their expenses. Our understanding is that these funds came from the care providers themselves and not the programme.

TB treatment can be lengthy and isolating. Treatment providers responded to patients’ psychosocial needs by providing activities and personalized counselling. At the SMRU TB village, staff set up handicrafts and gardening activities to boost patients’ spirits. WVT volunteers described ‘encouraging patients not to give up’ as part of their role. Patients explained that in some cases they were separated from their families during treatment and that they really valued the encouragement provided by health care providers. Treatment providers emphasized the importance of providing personalized counselling and described talking at length with patients about TB progression and the importance of adhering to treatment. A TB physician described a typical conversation, ‘You need to take treatment. It might take six months. So where would you like to get the treatment? Wherever you go the treatment is free of charge. The main thing is to select a location where you can get treatment until the end of the period.’

Providers explained that counselling around adherence was linked to concerns that high mobility among migrants might lead to treatment default and subsequent drug resistance. We found that mobility itself is an indirect barrier as it limits patients’ eligibility for care. Health care providers responded to mobility-related apprehensions by asking patients to commit to completing treatment and counselling individuals on available treatment locations. At the time we collected data, cross-border TB referral was being developed. A TB clinician expressed, ‘So once it’s diagnosed we want to at least reach them for counselling. Where do they want to take treatment? Myanmar or where?’

#### Reaching out to potential patients

Migrant TB patients described delaying seeking treatment until they could no longer work. Both WVT and SMRU TB programmes incorporated initiatives designed to reach out in search of potential TB patients.

In the 2 years prior to our data collection, WVT and SMRU introduced enhanced TB screening programmes. WVT changed from screening symptomatic cases to a community wide screening protocol. SMRU initiated screening of family contacts who were living in Thailand, in addition to members who accompanied the patient to the clinic. Enhanced TB screening can help identify asymptomatic TB cases and provide individuals with the opportunity to initiate early treatment and to avoid the negative consequences associated with late diagnosis.

In efforts to reach more patients, SMRU also set up a partnership with a popular migrant health centre, the MTC. SMRU TB doctors travel to MTC to diagnose potential TB patients who are then referred to the TB village. Additionally, SMRU also began offering HIV treatment to immediate family members of TB-HIV patients, who would otherwise be ineligible for free HIV treatment in the region. This programmatic change was made principally to prevent domestic abuse experienced by women who disclosed their HIV status to their husband.

### Additional responsive actors

Although we profile two TB treatment programmes in this article, it is important to note that there are other actors who have responded to migrants’ barriers to TB treatment. The first are migrants themselves. CHVs described how they had developed a community fund to collect money for transportation. This was initially a WVT project that is now run directly by the migrants. A volunteer explained, ‘We organized a volunteer group in our community and collect 20 baht/month (USD 0.60) from each member and save it to help our migrant workers when needed.’

Another volunteer detailed the purpose of these actions,

Our main point is to help our Burmese people to unite together and lift them up. Even though we are in another country, we don’t want people to look down on us, on our people. We plan and prepare for our people in case they need help. If they are sick and have to go to the hospital and they don’t have money for transportation fees then here is the money. We planned and saved it in advance. But we can’t pay for the treatment fees, we can just help with anything we can afford.

#### CHV survey

CHVs affiliated with the WVT TB programme are foreign labour migrants living at the edge of the urban core in neighborhoods that are densely populated by migrants. In each community, volunteers run a local health post where they provide health information, collect sputum for TB testing and help link migrants to TB care. Our survey with migrant CHVs enabled us to document their contributions to TB treatment, referral and surveillance. Table 2 provides information on the sample’s demographic characteristics. Participants were predominantly female (70%) with a majority aged 30–49 (62%). Another study from two Thai border provinces similarly found that most (56.9%) of the migrant volunteers were women (Sirilak *et al.* 2013). In our sample, most CHVs spoke Burmese, self-identified as documented migrants (60%), and had been working as a CHV for 2 or more years (54%).

In the 12 months prior to our survey in 2015, most CHVs had provided TB treatment (64%) and supervised directly observed treatment (DOT) (57%). A smaller percentage of respondents provided TB/HIV treatment (20%) and TB/HIV DOT (22%) in the same period. Few participants had provided HIV treatment (16%), MDR-TB treatment (10%) or supervised MDR-TB DOT (8%). Table 3 details other CHV contributions to treatment and surveillance.

**Table 3.** CHV contribution to TB treatment and surveillance

	*n* (%)
**Total**	101 (100%)
Contribution to treatment	
Provided TB treatment in last 12 months	65 (64%)
Supervised DOT in last 12 months	58 (57%)
Missing	1 (1%)
Provided MDR-TB treatment in last 12 months	10 (10%)
Provided TB/HIV treatment in the last 12 months	20 (20%)
Referral TB patients for HIV test (last 12 months)	
Never	30 (30%)
Rarely	2 (2%)
Sometimes	28 (28%)
Most of the time	21 (21%)
Always	20 (20%)
Referred TB patients for HIV testing to^a^	
Community health worker	24
World Vision	35
MTC	8
Hospital	8
Other	3
Not relevant	30
Missing	2
Referral of suspected TB patients for testing (last 12 months)	
Never	3 (3%)
Rarely	1 (1%)
Sometimes	22 (22%)
Most of the time	28 (28%)
Always	45 (45%)
Missing	2 (2%)
Contribution to Surveillance	
Notification of new suspected TB case	88 (87%)
Missing	1 (1%)
Organization notified re: suspected TB case	
Community health worker	26
Thai public health clinic	1
World Vision	56
Other	6
Not relevant	12
Missing	7
Collected data on infectious disease in the last 12 months	45 (45%)
Missing	1 (1%)

a Percentages are not reported for multiple answer questions.

To examine CHVs contribution to the identification of new TB and HIV cases we asked how often they had referred suspected cases for testing in the previous year. The majority of the respondents referred suspected TB patients for testing most of the time (28%) or always (45%) and indicated that they would notify someone if they found a new suspected case of TB (87%). Most respondents (56 individuals) would notify the NGO running the TB programme. Referral of TB patients for HIV testing was more evenly distributed between categories with never (30%), rarely (2%), sometimes (28%), most of the time (21%) and always (20%). In regard to surveillance in the previous year, 45% of participants had collected data on infectious disease in their catchment area.

## Discussion

Studies on the support provided to TB patients are limited (Noyes and Popay 2007). Along the Thailand–Myanmar border, TB treatment is standardized. Thai government hospitals follow national protocols and SMRU utilizes World Health Organization (WHO) recommendations for TB care. Success rates are close to global standards. SMRU’s TB treatment success rates for migrant patients in 2013 and 2014, 82%, were close to the WHO 85% success target rate (Tak Tuberculosis Border Initiative 2015). Our results suggest that in this context two NGOs have developed responsive and flexible programmes that are adapted to suit migrant patient’s needs. For example, after completing an initial residential period, migrants who are living in the Mae Sot area can choose community-based TB care or residential care. A strength of these programmes is that they help migrants overcome mobility challenges related to their legal status by simultaneously bringing patients into clinical spaces to receive care from physicians and also taking TB care out into the community. By providing free treatment both programmes address some of the catastrophic patient costs associated with TB treatment (Munro *et al.* 2007; Rasanathan *et al.* 2011). Giving patients the opportunity to continue working and receive care at home, or stay in a residential facility where food and accommodation are provided further diminishes their out of pocket expenditures. These programmes have incorporated the essence of migrant sensitive health service delivery through the provision of care in languages patients speak by community health workers and volunteers who are knowledgeable about the local culture.

In examining the actors who responded to address barriers to treatment, we found that migrants themselves were often frontline TB treatment providers. Although both of the TB programmes that we profile in this article were overseen by NGOs, TB care was provided by Thai and Burmese physicians with the assistance of migrant CHVs and medics. We perceive that medics’ and volunteers’ identities as migrants may further enhance their desire to respond to patient needs as a means to improve the lives of migrants living in Thailand. The way that CHVs framed their rationale for volunteering may be related to the way they see themselves, as members of a minority group in a foreign country where they need to work together in solidarity. Our survey did not collect information on reasons for volunteering but a CHV survey with a similar migrant population found that 98.1% believed that volunteer work assisted members of their ethnic group (Sirilak *et al.* 2013). This model of care, where migrants are embedded in treatment programmes as medics and volunteers, is not limited to TB programmes but is commonly used by numerous organizations that provide healthcare to migrant populations along the border. We perceive that this model is in itself responsive and that further research on health systems response for other health conditions is warranted.

Although we cannot quantify the impact of CHV work on TB control in Tak province, their contribution should not be ignored. Delayed care seeking is a barrier to TB treatment among migrant populations in this region (Tschirhart *et al.* 2016a). We found that 87% of CHV would notify someone if they found a suspected TB case, and we anticipate that this could lead to earlier access to treatment and a reduction of co-morbidities associated with delayed care seeking. In addition, CHVs’ provision of TB treatment in the community is likely to have positive economic spin-offs as migrants can receive care while continuing to work. Despite the potential contribution of CHVs to TB control, we anticipate that additional research is necessary to further explore migrants’ motivations for volunteering and the long-term sustainability of a volunteer run TB initiative. As volunteers CHVs are not paid. Identifying ways to compensate these individuals for their important work in supporting TB patients, identifying new cases, and contributing to the programme appears warranted.

From a policy and practitioner perspective it is important to note that many of the barriers that migrants reported exist outside of the health care system. Legal status, transportation and patient financial difficulties do not originate in the health system but are related to the social, legal and economic environments where migrants live (Tschirhart *et al.* 2016a). In responding to patient barriers to treatment, through the provision of accommodation, food and transport treatment providers are intervening to address the underlying social determinants of health. Health system actors described interceding with actions to improve migrants’ living conditions for the express purpose of improving access to TB treatment and adherence. This type of engagement was described as necessary and may point to a need for future inter-sectoral action to address TB in migrant populations along the Thailand–Myanmar border.

Our study found that significant financial and non-financial supports are necessary to help improve migrants’ access and adherence to TB treatment. Although this research is specific to the Thailand–Myanmar border, our results engage with broader issues of health equity and the inputs that are necessary to close the gap (Marmot 2015). Our results suggest that migrants need to be provided with resources that help stabilize their financial situation and overcome difficulties associated with their legal status in order to access and continue TB treatment. We agree with Richter *et al.* (2014) that cost-effectiveness is an important topic for future research as additional information is needed to improve the evidence base for policy decision-making.

Recent discussions on TB control among migrant populations in Europe have emphasized the potential benefits of pre-screening migrants from high burden countries (Aldridge *et al.* 2014, 2016). However, we agree with van der Werf and Kramarz (2016) that relying predominantly on pre-screening would overlook the undocumented migrants who arrive in their destination country without the appropriate visa. Based on our findings along the Thailand–Myanmar border we recommend countries consider how to make their TB screening programmes responsive to migrants needs. Provision of post-arrival screening free of charge to undocumented migrants, as is done in Israel, may be a beginning but national TB programmes may also wish consider how they can create programmes that allow migrants to receive screening without repercussion regardless of their legal status (Chemtob *et al.* 2015). In parallel to screening programmes, we believe that broader national TB programme migrant strategies should consider how they can incorporate migrants as health workers and liaisons between the public health department and the community.

When we were collecting the data for this project the national Thai government was implementing changes to the community migrant health insurance scheme. Previously, employers had to register migrants for this benefit but as of August 2014 migrants could register themselves and pay directly into this scheme (Prevention of HIV/AIDS Among Migrant Workers in Thailand 2014; Guinto *et al.* 2015). We interpret this policy measure as an example of responsiveness by the Thai government to the general difficulties that migrants have accessing healthcare through the public system in Thailand. However, we anticipate that many migrants cannot afford to buy into the migrant health insurance scheme.

In conclusion, we found two NGO TB programmes in Tak province that are highly responsive to the challenges migrants face when seeking treatment. Their responses necessarily extend beyond the health system and address the social, legal and economic environments where migrants live. This study from the Thailand–Myanmar border offers valuable lessons to policy makers and practitioners working with migrants in other jurisdictions. Organizations may benefit from identifying treatment barriers in consultation with patients and responding with initiatives that stabilize migrants’ financial situation and address obstacles associated with their legal status.

### Limitations

It is important to note that we did not collect information on the cost or effectiveness of these supportive measures. We collected our data over a 3-month period in 2014 and acknowledge that TB control and migrant health initiatives continue to evolve. As such, the results of our study should be considered within this temporal period. Our data, given its qualitative nature, cannot be generalized to the experiences of all migrants along the Thailand–Myanmar border. However, we anticipate that the themes generated by this work can contribute to the dialogue on migration and health system response in Southeast Asia and other regions such as Europe that are developing strategies to address TB control among migrants.
